# ST6GALNAC1 plays important roles in enhancing cancer stem phenotypes of colorectal cancer via the Akt pathway

**DOI:** 10.18632/oncotarget.22545

**Published:** 2017-11-08

**Authors:** Tadashi Ogawa, Yoshihiko Hirohashi, Aiko Murai, Toshihiko Nishidate, Kenji Okita, Liming Wang, Yuzuru Ikehara, Tetsuta Satoyoshi, Akihiro Usui, Terufumi Kubo, Munehide Nakastugawa, Takayuki Kanaseki, Tomohide Tsukahara, Goro Kutomi, Tomohisa Furuhata, Koichi Hirata, Noriyuki Sato, Toru Mizuguchi, Ichiro Takemasa, Toshihiko Torigoe

**Affiliations:** ^1^ Department of Pathology, Sapporo Medical University School of Medicine, Chuo-Ku, Sapporo 060-8556, Japan; ^2^ Department of Surgery, Sapporo Medical University School of Medicine, Chuo-Ku, Sapporo 060-8556, Japan; ^3^ The Molecular Medicine Team, Research Center for Medical Glycoscience, National Institute of Advanced Industrial Science and Technology, Tsukuba 305-8568, Japan

**Keywords:** colorectal cancer, cancer stem cell, ST6GALNAC1, STn antigen, Akt pathway

## Abstract

Colorectal cancer (CRC) is a mortal disease due to treatment resistance, recurrence and distant metastasis. Emerging evidence has revealed that a small sub-population of cancer cells termed cancer stem cells (CSCs)/ cancer-initiating cells (CICs) is endowed with high levels of tumor-initiating ability, self-renewal ability and differentiation ability and is responsible for treatment resistance, recurrence and distant metastasis. Eradication of CSCs/CICs is essential to improve current treatments. However, the molecular mechanisms by which CSCs/CICs are maintained are still elusive. In this study, we aimed to determine the molecular mechanisms by which colorectal (CR)-CSCs/CICs in are maintained human primary CRC cells. CR-CSCs/CICs were isolated by sphere-culture and the ALDEFLUOR assay, and transcriptome analysis revealed that the gene ST6 N-Acetylgalactosaminide Alpha-2,6-Sialyltransferase 1 (ST6GALNAC1) was expressed at high levels in CR-CSCs/CICs. Overexpression of ST6GALNAC1 enhanced the expression of sialyl-Tn (STn) antigen, which is carried by the CSC marker CD44, and increased the sphere-forming ability and resistance to a chemotherapeutic reagent. The opposite phenomena were observed by gene knockdown using siRNA. Furthermore, the Akt pathway was activated in ST6GANAC1-overexpressed cells, and activation of the pathway was cancelled by gene knockdown of galectin-3. The results indicate that ST6GALNAC1 has a role in the maintenance of CR-CSCs/CICs by activating the Akt pathway in cooperation with galectin-3 and that ST6GalNAC1 (or STn antigen) might be a reasonable molecule for CSC/CIC-targeting therapy.

## INTRODUCTION

Colorectal cancer (CRC) is one of the most common malignancies in the world [1], and the morbidity and mortality of CRC have been increasing in Japan. Recently, the survival of patients with CRC has been improved due to the development of sensitive diagnostic modalities and improvements of surgical techniques and treatments using anti-cancer agents, irradiation and other methods. However, the outcomes for patients with CRC who show treatment resistance, recurrence or distant metastasis remain poor.

Cancer tissues are composed of a heterogeneous subpopulation of cancer cells, and small sub-populations are endowed with tumor-initiating ability. Highly tumorigenic cancer cells are called cancer stem-like cells (CSCs)/ cancer-initiating cells (CICs) [2]. CSCs/CICs are defined as cells with the following properties: self-renewal ability, ability to differentiate into multiple cell types, and high levels of tumorigenicity and therapeutic resistance [3]. Recent studies have shown that CSCs/CICs are resistant to standard cancer therapies [4], and it has been shown that CSCs/CICs are correlated to recurrence and distant metastasis, which are lethal events for cancer patients [5–7]. Moreover, recurrent or metastatic lesions acquire tolerance to anti-cancer agents due to replacement of cell populations by CSC/CIC-rich populations caused by repeated treatments [8]. Therefore, eradication of CSCs/CICs is essential to cure cancer, and identification of molecular targets for CSC/CIC-targeting therapy is needed.

Altered glycosylation can be observed in cancer cells, and overexpression of sialylated antigens on the surface of cancer cells has been reported [9, 10]. The sialyl-Tn (STn) antigen (Neu5Acα2-6Gal-NAcα1-O-Ser/Thr), also known as CD175s, is a mucin-type carbohydrate antigen, and STn antigen is aberrantly expressed in a variety of carcinomas, including gastric [11, 12], colorectal [13], ovarian [14], breast [15], and pancreatic [16] cancers. STn antigen expression has been reported to be related to cancer invasion and metastasis [17–19]. However, there has been no report in which the relationship between STn antigen and cancer stem cells is described.

In this study, we isolated colorectal cancer stem-like cells/ cancer initiating cells (CR-CSCs/CICs) from freshly resected colon cancer tissue. The transcriptome of CR-CSCs/CICs was analyzed by using a cDNA microarray, and we identified ST6GALNAC1 as a candidate of CR-CSC/CIC-specific genes. The cancer-associated Sialyl-Tn (STn) antigen is a short O-glycan containing a sialic acid residue in an α2,6-linkage to GalNAc α-O-Ser/Thr, and biosynthesis of the STn antigen is mediated by ST6GALNAC1 specifically [20]. The STn antigens are not expressed or are expressed at very low levels in normal adult tissues; however, the STn antigens are expressed at high levels in adenocarcinomas [21]. STn antigen expression is known to be correlated with cancer aggressiveness and poor prognosis, but there have been few reports about the relationship between CSCs/CICs and STn antigens. We analyzed the relationship between expression of ST6GALNAC1 in CR-CSCs/CICs and the potential molecular mechanisms by which of CR-CSCs/CICs are maintained.

## RESULTS

### Isolation of CR-CSCs/CICs from human primary CR tissues

**Figure 1:**
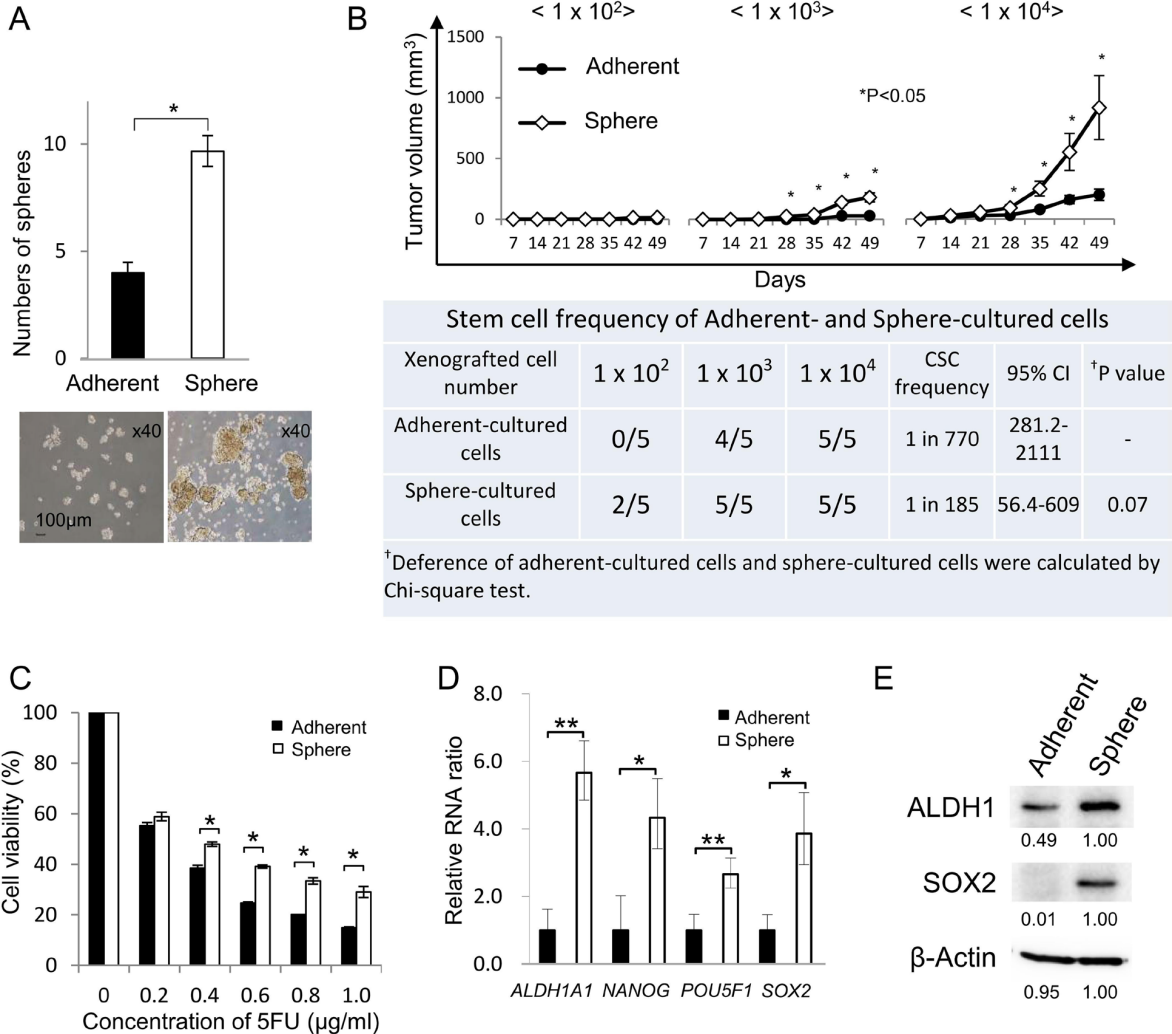
Isolation of CR-CSCs/CICs from human primary CRCs (**A**) Sphere-forming ability of sphere-cultured cells and adherent-cultured cells. Human primary colorectal cancer cells (CRC21) were cultured in serum-free sphere culture and serum adherent culture conditions. The sphere-forming ability of sphere-cultured cells and adherent-cultured cells was examined. Spheres derived from 2500 adherent-cultured cells and from 2500 sphere-cultured cells were counted. Data are shown as means ± SD. An asterisk indicates statistical difference. Representative images of spheroids derived from adherent- and sphere-cultured cells are shown. (**B**) *In vivo* tumorigenicity of sphere-cultured cells and adherent-cultured cells. Upper panel: Tumor growth of adherent-cultured cells and sphere-cultured cells. 1.0 × 10^2^, 1.0 × 10^3^ and 1.0 × 10^4^ cells were injected in the middle backspace of each of the recipient mice under anesthesia and tumors were monitored every week until 7 weeks after injection (*n* = 5). Data are shown as means ± SD. An asterisk indicates statistical difference. Lower table: Summary of tumor initiation. Estimated CSC ratio was calculated by the ELDA web site. CI: confidence interval. (**C**) Resistance to 5-FU. Adherent- and sphere-cultured cells were incubated in a culture medium containing 5-FU at several concentrations for 4 days. Cell viability was examined by the WST-1 assay. Data are shown as means ± SD. An asterisk indicates statistical difference. (**D**) Quantitative RT-PCR analysis of stem cell-related gene expression in sphere-cultured cells and adherent-cultured cells. The expression levels of ALDH1A1, NANOG, POU5F1 and SOX2 were examined by qRT-PCR using cDNAs derived from adherent-cultured cells and sphere-cultured cells. Data are shown as means ± SD. An asterisk indicates statistical difference. (**E**) Western blot analysis of ALDH1 and SOX2. The protein expression of ALDH1 and SOX2 was examined using adherent-cultured cells and sphere-cultured cells. Numerical data indicate relative intensity of the bands determined by ImageJ software. β-Actin was used as an internal control.

Methods for isolating human CR-CSCs/CICs include isolation using CSC/CIC markers (CD133, CD44), side population assay, ALDEFLUOR assay and sphere culture [22–26]. In this study, we used sphere culture and ALDEFLUOR assay to obtain primary human CR-CSCs/CICs. We performed primary cultures from 50 freshly isolated human CRC tissues to obtain cancer cells that can grow stably *ex vivo* under adherent culture and sphere culture conditions, and we succeeded in obtaining primary CRC cells from one case (case #21: 75-year-old female patient with ascending colon carcinoma). The primary CRC cells were termed CRC21 cells. A sphere-forming assay revealed that sphere-cultured cells formed a larger number of spheroids and spheroids of larger sizes than did adherent-cultured cells (Figure 1A). A xenograft transplantation in immune-deficient mice was performed, and the estimated stem cell ratio in sphere-cultured cells was 1 in 185 cells, whereas the estimated stem cell ratio in adherent-culture cells was 1 in 770; however, the difference in stem cell ratios did not reach statistical significance (Figure 1B, *P* = 0.07). Tumors derived from injections of 1.0 × 10^3^ and 1.0 × 10^4^ sphere-cultured cells showed significantly higher rates of growth than did those derived from adherent-cultured cells (Figure 1B upper panel and Supplementary Figure 1A and 1B). Tumor initiation was observed in 2 of 5 mice that were injected with 1 × 10^2^ sphere-cultured cells, but tumor initiation was not observed when they were injected with 1 × 10^2^ adherent-cultured cells (Figure 1B). Sphere-cultured cells showed higher resistance against 5-FU (Figure 1C) and higher expression levels of stem cell-related genes (ALDH1A1, NANOG, POU5F1 and SOX2) than did adherent-cultured cells (Figure 1D). Sphere-cultured cells showed higher levels of ALDH1 and SOX2 protein expression than the levels in adherent-cultured cells (Figure 1E). Sphere-cultured cells showed higher expression levels of CD44 and CD133 (Supplementary Figure 2). These results suggested that CR-CSCs/CICs were enriched in sphere-cultured cells.

**Figure 2:**
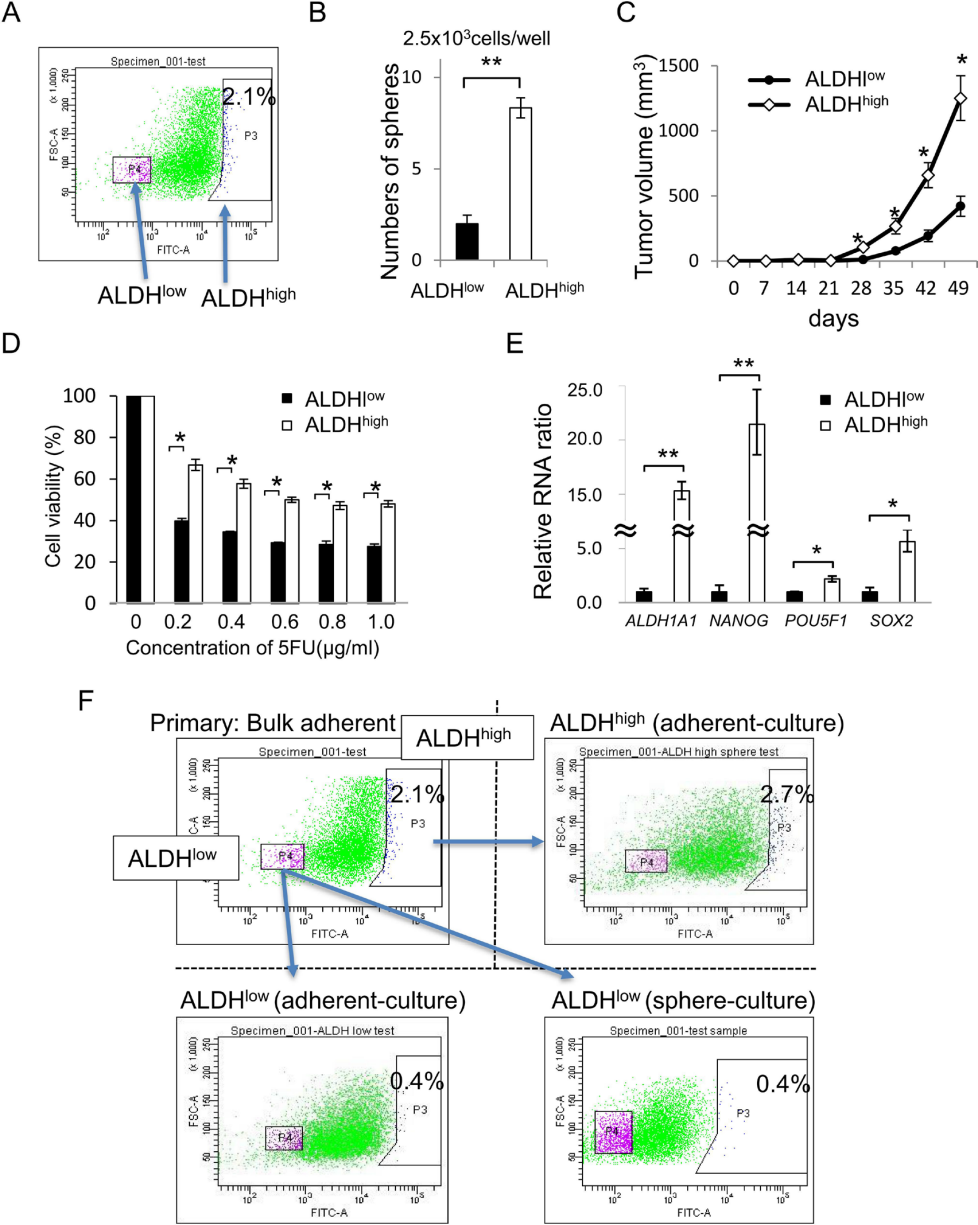
ALDHhigh cells have a character of CR-CSCs/CICs (**A**) ALDEFLUOR assay using adherent-cultured cells. Results of an ALDEFLUOR assay using adherent-cultured CRC21 cells are shown. ALDH^high^ cells were defined by DEAB, an ALDH inhibitor. The ratio of ALDH^high^ cells was 2.1%. (**B**) Sphere-forming assay using ALDHhigh cells and ALDHlow cells. Numbers of spheres from 2500 ALDH^high^ cells and 2500 ALDH^low^ cells are shown. Data are shown as means ± SD. An asterisk indicates statistical difference. (**C**) *In vivo* tumorigenicity of ALDHhigh cells and ALDHlow cells. Four thousand ALDH^high^ cells and 4000 ALDH^low^ cells were injected into NOD/SCID mice. Tumors were monitored every week until 7 weeks after injection. Data are shown as means ± SD. An asterisk indicates statistical difference. (**D**) Resistance to 5-FU. ALDH^high^ cells and ALDH^low^ cells were incubated in culture media containing 5-FU at several concentrations for 4 days. Cell viability was examined by the WST-1 assay. Data are shown as means ± SD. An asterisk indicates statistical difference. (**E**) Quantitative RT-PCR analysis of stem cell-related gene expression in ALDHhigh cells and ALDHlow cells. Expression levels of ALDH1A1, NANOG, POU5F1 and SOX2 were examined by qRT-PCR. Data are shown as means ± SD. An asterisk indicates statistical difference. (**F**) Self-renewal and differentiation of ALDHhigh cells and ALDHlow cells. ALDH^high^ and ALDH^low^ cells were isolated from adherent-cultured cells. ALDH^high^ cells were cultured in an adherent condition. ALDH^low^ cells were cultured in an adherent condition and sphere condition. The ratio of ALDH^high^ cells in adherent-cultured ALDH^high^ cells was 2.7%. The ratios of ALDH^high^ cells in adherent- and sphere-cultured ALDH^low^ cells were 0.4%.

In the following experiments, we performed the ALDEFLUOR assay using adherent-cultured cells to enrich CR-CSCs/CICs. Cells with a high level of aldehyde dehydrogenase activity (ALDH^high^ cells) were detected in adherent-cultured cells, and the ratio of ALDH^high^ cells was 2.1% (Figure 2A). A sphere-forming assay revealed that ALDH^high^ cells formed significantly greater numbers of spheroids than did cells with a low levels of aldehyde dehydrogenase activity (ALDH^low^ cells) (Figure 2B). A xenograft transplantation assay using 4000 ALDH^high^ cells and ALDH^low^ cells revealed that tumors derived from ALDH^high^ cells showed greater growth than did tumors derived from ALDH^low^ cells (Figure 2C). ALDH^high^ cells showed higher resistance to 5-FU and higher expression levels of stem cell-related genes (ALDH1A1, NANOG, POU5F1 and SOX2) than did ALDH^low^ cells (Figure 2D and 2E). We cultured isolated ALDH^high^ cells and ALDH^low^ cells and analyzed the cells by an ALDEFLUOR assay again. Adherent-cultured ALDH^low^ cells showed a lower ratio of ALDH^high^ cells than that in adherent-cultured ALDH^high^ cells. The ratio of ALDH^high^ cells in isolated ALDH^low^ cells was not increased by sphere culture (Figure 2F). These observations suggested that CR-CSCs/CICs were enriched in ALDH^high^ cells and that ALDH^high^ cells can differentiate into ALDH^low^ cells; however, ALDH^low^ cells cannot de-differentiate into ALDH^high^ cells.

### Identification of ST6GALNAC1 as a CR-CSC/CIC-specific gene

**Figure 3:**
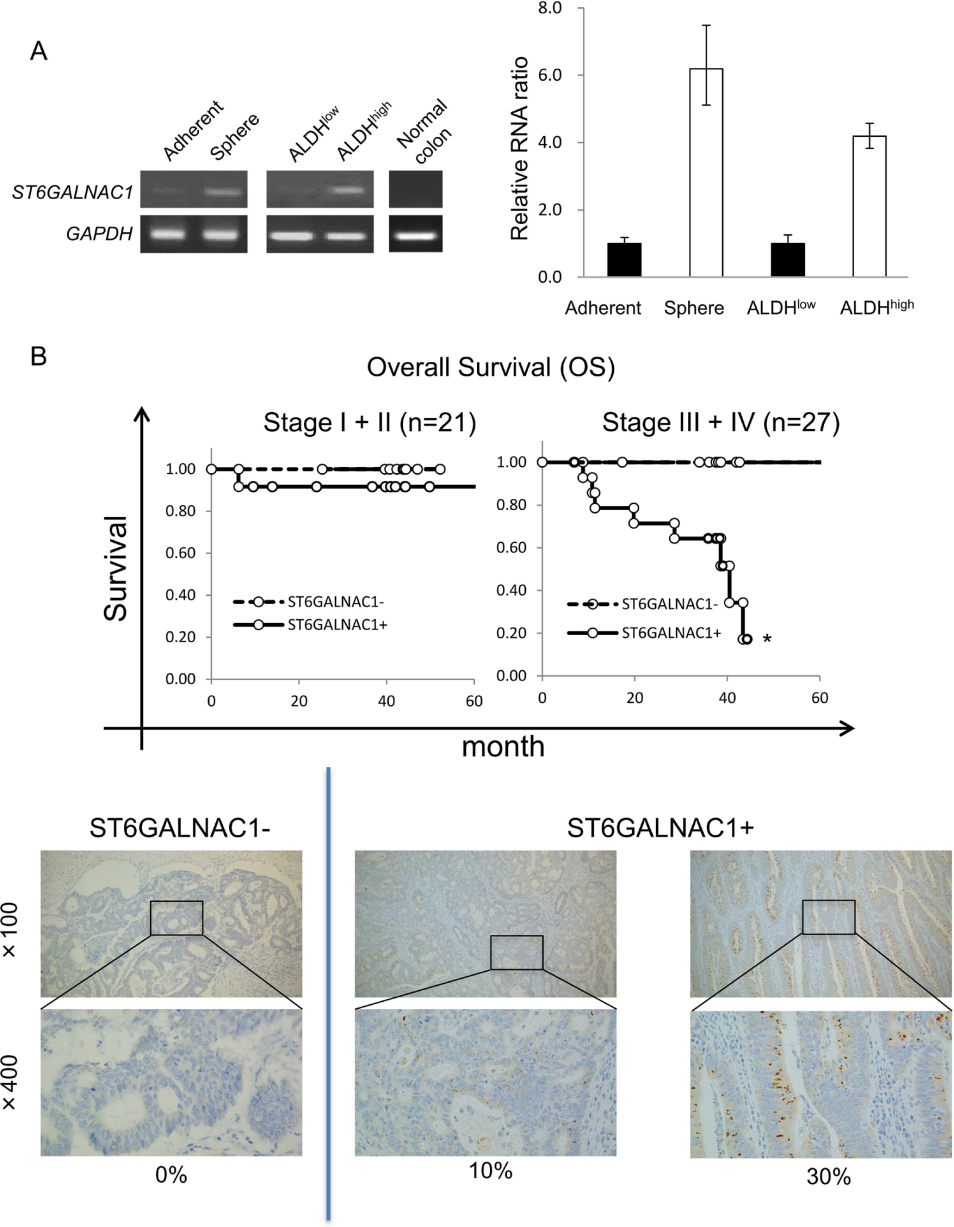
Expression of ST6GALNAC1 in CR-CSCs/CICs and clinical samples (**A**) RT-PCR and qRT-PCR analyses of ST6GALNAC1 in CR-CSCs/CICs, non-CSCs/CICs and normal colon mucosa. Expression of *ST6GALNAC1* was analyzed by RT-PCR using cDNAs derived from adherent-cultured cells, sphere-cultured cells, ALDH^high^ cells, ALDH^low^ cells and normal colon tissue (left). *GAPDH* was used as an internal control. Expression of *ST6GALNAC1* was analyzed by qRT-PCR using cDNAs of adherent-cultured cells, sphere-cultured cells, ALDH^high^ cells and ALDH^low^ cells. Data are shown as means ± SD. An asterisk indicates statistical difference. (**B**) Overall survival (OS) for patients with ST6GALNAC1-positive cancer and those with ST6GALNAC1-negative cancer after primary surgery. ST6GALNAC1 protein was detected by immunohistochemical (IHC) staining using an ST6GALNAC1-specific antibody in colorectal cancer cases. The positive rates of ST6GALNAC1 were 0%–80%. ST6GALNAC1-positive patients in Stage III and IV showed a significantly shorter survival time than that of ST6GALNAC1-negative patients in Stage III and IV after surgery (upper panel). Representative images are shown (lower panel). ST6GALNAC1-negative case (0%) and ST6GALNAC1-positive cases (10% and 30%). Magnification, ×100 and ×400.

To analyze CR-CSCs/CICs at the molecular level, we performed cDNA microarray analysis using CR-CSC/CIC samples including sphere-cultured cells and ALDH^high^ cells (summarized in Supplementary Figure 3). We found that 1220 genes were up-regulated in sphere-cultured cells compared with their expression levels in adherent-cultured cells and that 1531 genes were up-regulated in ALDH^high^ cells compared with their expression levels in ALDH^low^ cells. To obtain CR-CSC/CIC-specific gene candidates, we searched for overlapping up-regulated genes in sphere-cultured cells and ALDH^high^ cells and we found 245 candidates. To narrow down the candidate genes, we searched a public database and literature and we found 30 genes that may be related to cancer progression. We then performed RT-PCR to confirm the gene expression in CR-CSCs/CICs and non-CR-CSCs/CICs. Finally, we found that ST6GALNAC1 was expressed in a CSC/CIC sample (sphere-cultured cells and ALDH^high^ cells) but not in a non-CSC/CIC sample or normal colon tissue (Figure 3A). To generalize the expression of ST6GALNAC1 in CR-CSCs/CICs, we performed RT-PCRs using adherent-cultured cells and sphere-cultured cells of CR cell line cells HCT116 and SW480. ST6GALNAC1 was preferentially expressed in sphere-cultured cells rather than in adherent-cultured cells (Supplementary Figure 4). These results suggest that ST6GALNAC1 is a candidate of CR-CSC/CIC-specific genes.

Next, we performed statistical analysis to determine the clinical impact of ST6GALNAC1 by immunohistochemical staining (IHC) using an anti-ST6GALNAC1 antibody. The positive rates of ST6GALNAC1 were 0% to 80%, and we divided the cases into two groups: ST6GALNAC1-negative cases (ST6GALNAC1: 0%) and ST6GALNAC1-positive cases (ST6GALNAC1: 5%–80%). We analyzed overall survival (OS) after surgery for ST6GALNAC1-positive cases and negative cases, and we found that patients with ST6GALNAC1-positive cancer had a significantly shorter OS than that of patients with ST6GALNAC1-negative cancer for Stage III and IV, whereas no significant difference was found for Stage I and II (Figure 3B). These results suggested that expression of ST6GALNAC1 might be involved in cancer aggressiveness and poor prognosis.

### Role of ST6GALNAC1 in the maintenance of CR-CSCs/CICs by activating the Akt pathway via Galectin-3

**Figure 4:**
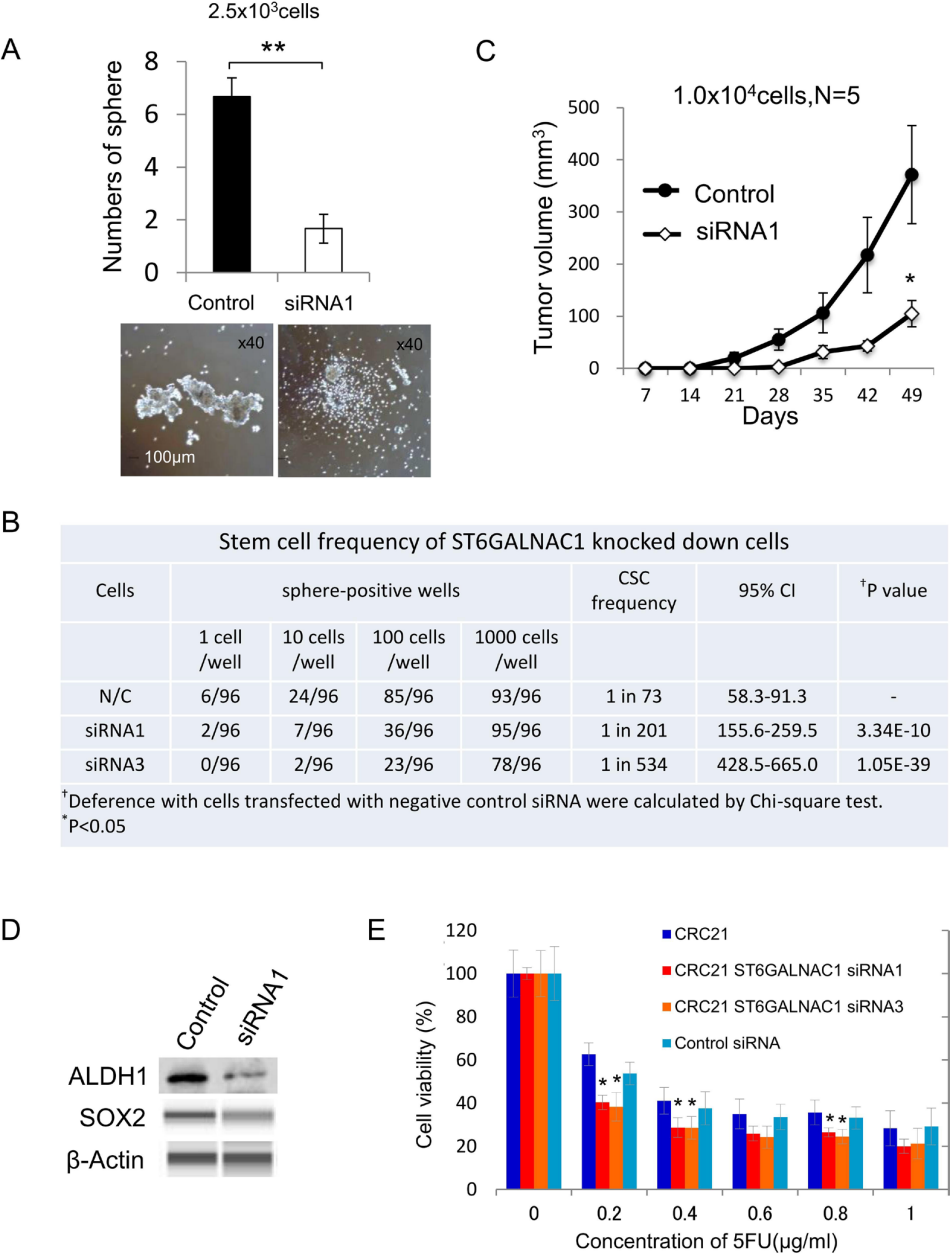
ST6GALNAC1 knockdown experiments using siRNAs (**A**) Sphere-forming ability of ST6GALNAC1-knockdown cells and negative control cells. Upper: Numbers of spheres from 2500 negative control cells and 2500 ST6GALNAC1-knockdown cells. Data are shown as means ± SD. An asterisk indicates statistical difference. Lower: Representative images of spheres derived from negative control cells and ST6GALNAC1-knockdown cells. Magnification, x 40. (**B**) Stem cell ratios in ST6GALNAC1-knockdown cells. CRC21 cells transfected with siRNAs (control, ST6GALNAC1 siRNA1, ST6GALNAC1 siRNA3) were seeded into a 96-well plate at 1, 10, 100 and 1000 cells/well under a sphere-forming condition and cultured for 14 days. The sphere-positive wells were counted and are shown. Stem cell ratios in negative control cells and ST6GALNAC1-knockdown cells were calculated by the ELDA website. (**C**) *In vivo* tumorigenicity of ST6GALNAC1-knockdown cells. Ten thousand ST6GALNAC1 siRNA-transfected CRC21 cells and 10,000 control siRNA-transfected CRC21 cells were injected into NOD/SCID mice (*n* = 5). The tumor growth was monitored every week until 7 weeks after injection. Data are shown as means ± SD. An asterisk indicates statistical difference. (**D**) Western blot analysis of ALDH1 and SOX2. The protein expression of ALDH1 and SOX2 was examined using ST6GALNAC1 siRNA-transfected adherent-cultured cells. The protein expression level of SOX2 was low in adherent cultured cells and the protein was detected by the WES system. β-Actin was used as an internal control. (**E**) Resistance to 5-FU. ST6GALNAC1 siRNA-transfected CRC21 cells were incubated in culture media containing 5-FU at several concentrations for 4 days. Cell viability was examined by the WST-1 assay. Data are shown as means ± SD. An asterisk indicates statistical difference.

The data of IHC staining suggested that ST6GALNAC1 might correlate with poor prognosis or aggressiveness. We therefore analyzed the relationship between ST6GALNAC1 and CSCs/CICs. ST6GALNAC1 gene expression in primary cultured CRC21 cells was knocked down by siRNAs. A qRT-PCR analysis revealed that CT6GALNAC1 expression was significantly decreased by siRNA1 and siRNA3 (Supplementary Figure 5). Suppression of the gene expression of ST6GALNAC1 resulted in less sphere-forming ability, suggesting a decrease in the proportion of CR-CSCs/CICs (Figure 4A). Thus, we analyzed the stem cell ratio of each sample by sphere-forming ability under a limiting dilution condition and analyzed the stem cell ratio by ELDA (Extreme Limiting Dilution Analysis) software. The CR-CSC/CIC ratio in negative control siRNA-transfected cells was 1 in 73 cells. On the other hand, the CR-CSC/CIC ratios in siRNA1-transfected cells and siRNA3-transfected cells were 1 in 201 and 1 in 534 (0.18%), respectively, and the difference in the CR-CSC/CIC ratios was statistically significant (*P* < 0.005) (Figure 4B). To further confirm the lower ratio of CR-CSC/CIC in ST6GALNAC1-knockdown cells, we performed xenograft transplantation. We injected 1.0×10^4^ cells with knockdown of ST6GALNAC1 by siRNA1 and the same number of negative control cells subcutaneously in recipient mice. ST6GALNAC1-knockdown cells formed significantly smaller tumors than did negative control siRNA-transfected cells (Figure 4C). ST6GALNAC1 knockdown using siRNA1 decreased the protein expression levels of ALDH1 and SOX2 (Figure 4D). ST6GALNAC1 knockdown significantly increased the sensitivitiy to 5-FU (Figure 4E). These results indicate that knockdown of ST6GALNAC1 decreased CR-CSCs/CICs. A decrease in the CR-CSC/CIC ratio by ST6GALNAC1 knockdown was observed using another CR cell line, HCT116 (Supplementary Figure 6), indicating that the relation of ST6GALNAC1 and CR-CSCs/CICs is a common molecular pathway.

**Figure 5:**
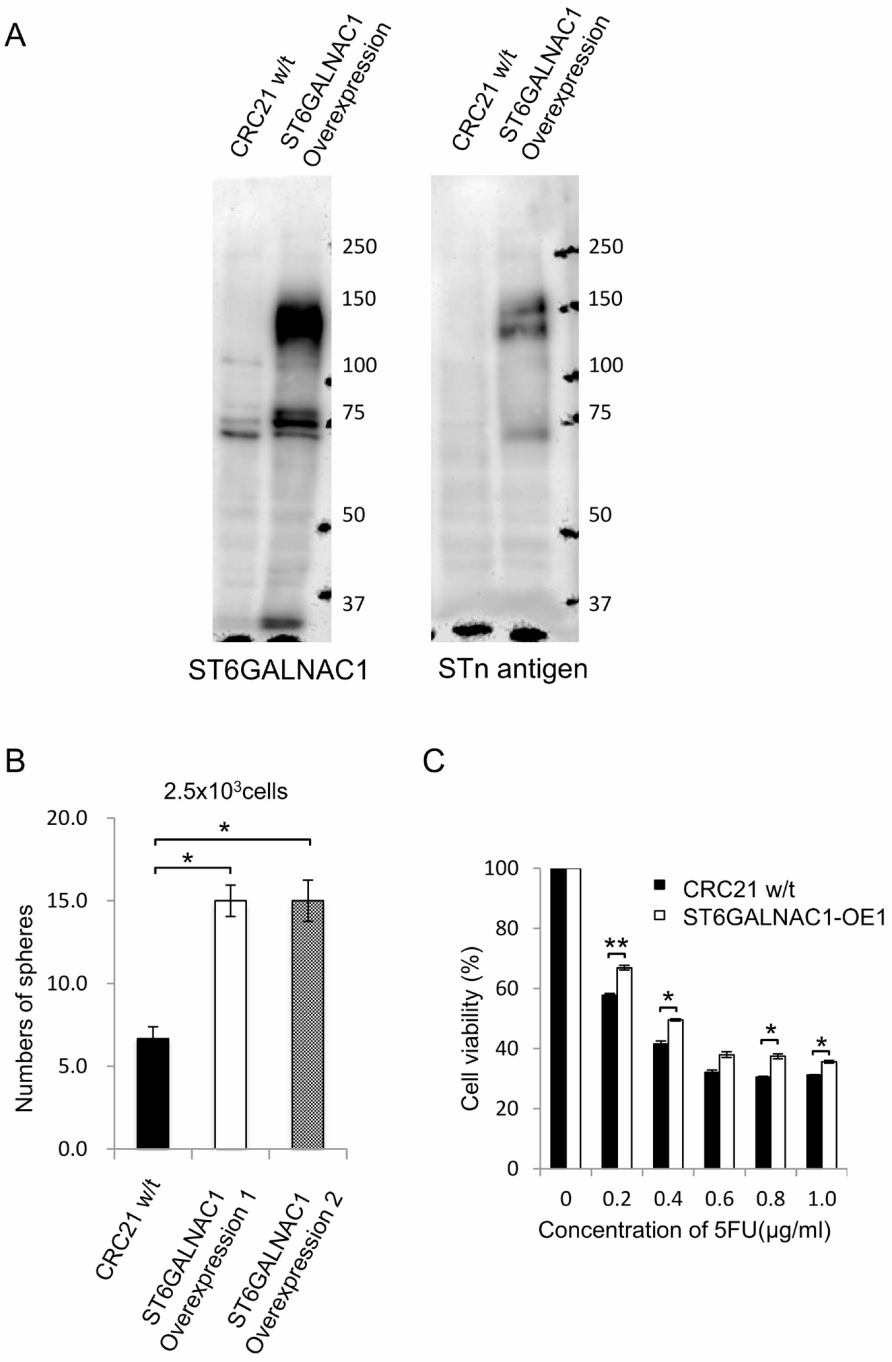
ST6GALNAC1 overexpression experiments (**A**) Western blot analysis of wild-type and ST6GALNAC1-overexpressed cells. Western blots were performed using anti-ST6GALNAC1 protein antibody and anti-STn antigen antibody. CRC21 wild-type cells and ST6GALNAC1-overexpressed clone 1 (OE1) and clone 2 (OE2) cells were used. β-Actin was used as an internal control. (**B**) Sphere-forming ability of ST6GALNAC1-overepxressed cells. Numbers of spheres from 2500 wild-type adherent-cultured cells and from 2500 each of two ST6GALNAC1-overexpressed cell clones (OE1 and OE2) are shown. Data are shown as means ± SD. An asterisk indicates statistical difference. (**C**) Resistance to 5-FU. ST6GALNAC1-overexpressed cells and control cells were incubated in culture media containing 5-FU at several concentrations for 4 days. Cell viability was examined by the WST-1 assay. Data are shown as means ± SD. An asterisk indicates statistical difference.

In the following experiments, we established ST6GALNAC1-overexpressed (OE) cells. We confirmed the expression of ST6GALNAC1 by RT-PCR (data not shown). Western blot analysis using anti-ST6GALNAC1 and anti-STn antigen revealed higher protein expression levels of ST6GALNAC1 and STn antigen in ST6GALNAC1-overexpressed cells than in control wild-type CRC21 cells (Figure 5A). The molecular weight of STn antigen was ~ 130 kDa. To clarify the carrier protein of STn antigen, we performed immunoprecipitation using an anti-STn antibody. Immunoprecipitated samples were analyzed by silver staining, and a specific band for ST6GALNAC1 overexpression was observed around 130 kDa. Western blot analysis using anti-STn antigen also revealed that a band around 130 kDa is specifically expressed in ST6GALNAC1-overexpressed cells. We therefore analyzed the band by mass spectrometry and identified the band as CD44. To confirm the information, we performed Western blot analysis using an anti-CD44 antibody. A specific band could be detected in ST6GALNAC1-overexpressed samples. Thus, CD44 is an STn antigen carrier protein (Supplementary Figure 7).

To address the functions of ST6GALNAC1 overexpression, a sphere-forming assay was performed and resistance to 5-FU was examined. A sphere-forming assay showed that ST6GALNAC1 OE cells made significantly larger numbers of spheres than did control bulk adherent cells (Figure 5B). ST6GALNAC1 OE1 cells showed higher resistance to 5-FU (Figure 5C). Taken together, the results obtained by using gene knockdown suggested that ST6GALNAC1 has a role in enhancing phenotypes of CR-CSCs/CICs.

**Figure 6:**
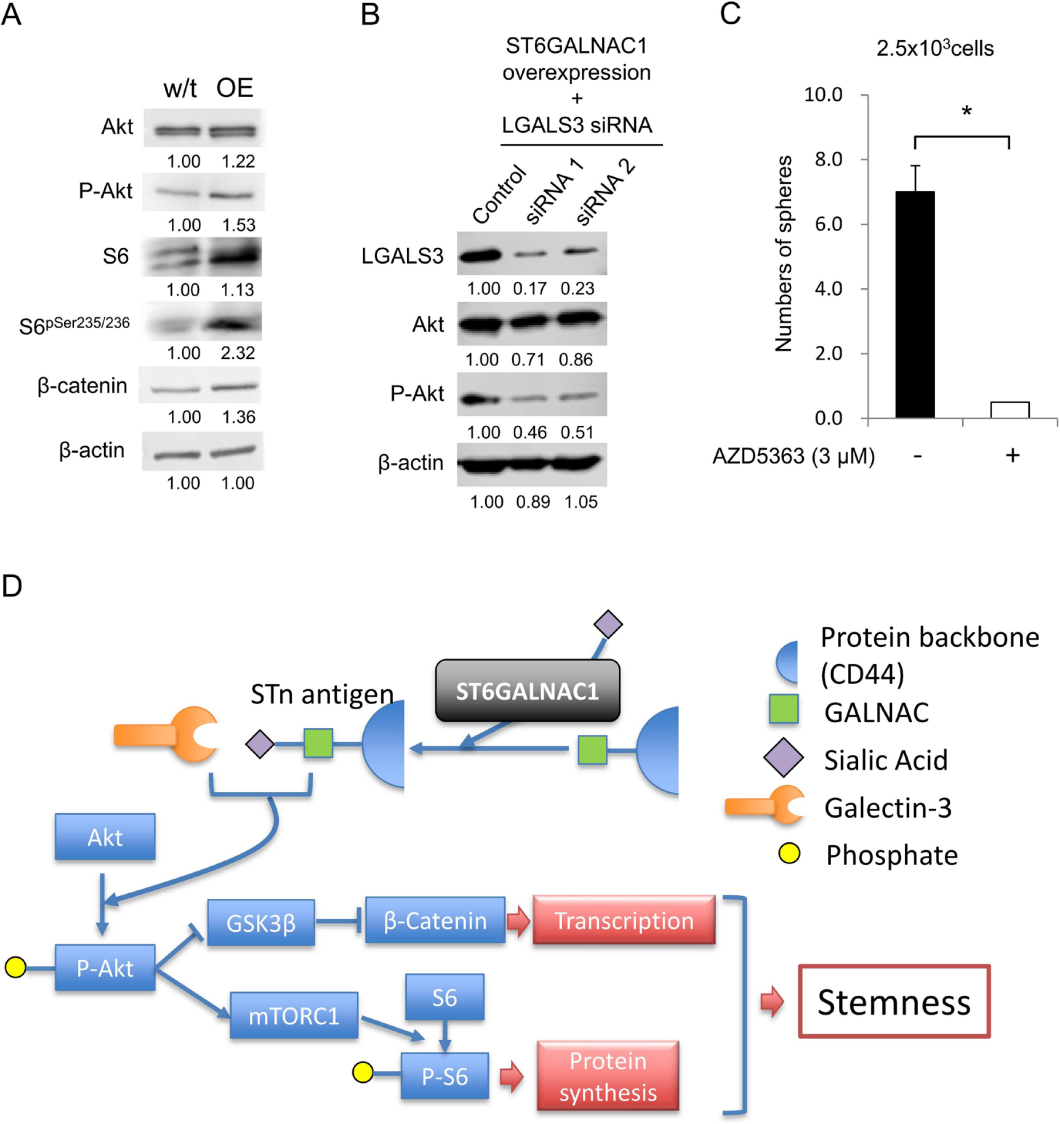
ST6GALNAC1 cooperates with galectin-3 to activate the Akt pathway (**A**) Western blot analysis of the Akt pathway. Activation of the Akt signaling pathway in wild-type CRC21 cells and ST6GALNAC1-overexpressed cells was examined by Western blot analysis using anti-Akt, phosphorylated Akt, S6, phosphorylated S6 and β-catenin antibodies. Numerical data indicate the relative intensity of bands (wild-type = 1). β-Actin was used as an internal control. (**B**) Phosphorylation of Akt under the condition of galectin-3 knockdown in ST6GALNAC1-OE cells. ST6GALNAC1-overexpressed CRC21 cells were transfected with LGALS3 (galectin-3) siRNAs. Knockdown of LGALS3 was confirmed by an Western blot analysis. Protein levels of Akt and phospho-Akt were determined Western blot analysis. Numerical data indicate the relative intensity of bands (control siRNA = 1). β-Actin was used as an internal control. (**C**) Sphere-forming ability of AZD5363-treated cells. 2.5 × 10^3^ CRC21 cells were cultured in a sphere condition including 3 μM of AZD5363. Data are shown as means ± SD. An asterisk indicates statistical difference. (**D**) Schematic summary of the relationship between STn antigen and Akt pathway. ST6GALNAC1 produces STn antigen by adding sialic acid onto GALNAL and the protein backbone. Intracellular STn antigen binds and activates galectin-3. Activated gelactin-3 promotes phosphorylation of Akt. Phosphorylated Akt suppresses GSK3β and then increases transcription by β-catenin. Phosphorylated Akt increases S6 phosphorylation by mTORC1 and then increases protein synthesis. Finally, the cell obtains stemness by activated transcription and protein synthesis.

A previous study revealed the expression of STn antigen and activation of PI3K/Akt signaling [27], and we thus analyzed the Akt pathway by Western blot analysis. ST6GALNAC1 OE cells showed higher protein expression levels of phospho-Akt, S6^pser235/236^ and beta-catenin (Figure 6A), and these results suggested that STn antigen might be correlated with activation of the Akt pathway. In a previous study, the galectin-3 receptor for STn antigen was shown to have a role in activation of the PI3K/Akt pathway [28]. We therefore hypothesized that galectin-3 has a role in the PI3K/Akt pathway by binding STn antigen. To investigate the relation between STn antigen and galectin-3, we suppressed gene expression of galectin-3 (LGALS3) in ST6GALNAC1 OE cells by using siRNAs (Figure 6B). Western blot analysis revealed that the phosphorylation level of AKT at serine 473 residue was decreased by galectin-3 gene knockdown of ST6GALNAC1-overexpressed cells using galectin-3-specific siRNAs (Figure 6B). To confirm the relation of Akt signaling, an Akt inhibitor, AZD5363, was used. AZD5363 significantly decreased sphere formation and the expression levels of ALDH1A1 and SOX2 (Figure 6C and Supplementary Figure 8). These results suggest that STn antigen activates Akt by phosphorylation in cooperation with galectin-3 (Figure 6D).

## DISCUSSION

In the present study, we isolated CR-CSCs/CICs from human primary colorectal cancer tissues by using sphere culture and the ALDEFLUOR assay. Colorectal cancer cell lines are used worldwide as human disease models; however, some studies have shown that human cancer cell lines might have different genomic characteristics as in primary tumors [29]. Thus, CR-CSCs/CICs derived from primary cancer tissues are ideal sources to study human CR-CSCs/CICs. Sphere culture, the ALDEFLUOR assay and isolation using CD133 were previously used to isolate CR-CSCs/CICs. However, a controversial phenomenon regarding CD133 was described in a report [30]. Therefore, isolation of CR-CSCs/CICs should be validated by tumor initiation (tumorigenicity) using immune-deficient mice [3]. We confirmed the tumorigenicity of sphere-cultured cells and ALDH^high^ cells derived from primary tumors, and the cells used in this study are thus a reasonable source to analyze CR-CSCs/CICs. cDNA microarray analysis revealed that there are overlapped genes in sphere-cultured cells and ALDH^high^ cells; however, there are still difference between gene expression profiles in sphere-cultured cells and ALDH^high^ cells. These results indicate that both sphere-cultured cells and ALDH^high^ cells are enriched with highly tumorigenic CR-CSCs/CICs; however, there might be phenotypic difference.

The carbohydrate chain plays essential roles in post-translational protein processing and signal transduction, and it is closely associated with the maintenance of homeostasis of living organisms. Therefore, a deficit or alteration in the structure of the carbohydrate chain is known to be a cause of the development of diseases such as diabetes and pulmonary emphysema including malignant neoplasms. ST6GALNAC1 is a sialyltransferase that is expressed in the Golgi body and transfers sialic acid onto the O-linked sugar chain of the recipient protein backbone and makes sialyl-Tn antigen (STn antigen). STn antigen is overexpressed in some adenocarcinomas including colon, gastric, pancreas, breast, prostate and ovary adenocarcinomas, but it has limited or no expression in normal organs [31]. The functions of STn antigen are thought to be related to cell-to-cell attachment and cell migration, but recent studies have suggested associations with cancer aggressiveness and poor prognosis [32–34]. For example, Ozaki et al. reported that overexpression of the ST6GALNAC1 gene in a gastric cancer cell line enhanced metastatic ability [33], and Julien et al. reported that ST6GalNAC1-overexpressed breast cancer cell lines had enhanced tumorigenicity [32]. A recent study revealed that STn antigen expression is related to poorer prognosis in bladder cancer cases [27], and IHC analysis showed that the expression of STn antigen is related to activated PI3K/AKT/mTOR signaling. These results indicate that STn antigen has roles in cancer promotion by activation of Akt signaling; however, there is no direct evidence. In this study, we found that ST6GALNAC1 is preferentially expressed in primary CR-CSCs/CICs compared with its expression in non-CSCs/CICs. STn antigen has been reported to be abundantly expressed in most primary adenocarcinomas; however, the expression level of ST6GALNAC1 is often low in established long-cultured cell lines [20, 32–34]. Thus, the use of primary cancer cells is essential to analyze the molecular functions of ST6GALNAC1 and STn antigen.

In this study, we found a relationship between STn antigen and the Akt pathway. Overexpression of ST6GALNAC1 increased sialylation of CD44 antigen and increased the expression of STn antigen. STn antigen in cooperation with galectin-3 activated the Akt pathway. Activation of the Akt pathway increased transcription activity of β-catenin and protein synthesis by activation of S6 (Figure 6D). Previous studies also revealed that Akt signaling has roles in the maintenance of CR-CSCs/CICs [35, 36]. Galectin-3 is widely expressed in intracellular and extracellular spaces and it is a member of the family of carbohydrate-binding proteins associated with cell attachment, angiogenesis, cell proliferation and inhibition of apoptosis, which promotes cancer progression and metastasis [28, 37, 38]. We found that ST6GALNAC1 activates the Akt pathway via galectin-3. Thus, ST6GALNAC1 and its product STn antigen might be reasonable molecular targets for CR-CSC/CIC-targeting therapy. Todaro et al. also reported that Akt is acitivated by hepatocyte growth factor (HGF), osteopontin (OPN) and stromal cell-derived factor-1 (SDF-1) in CD44v6-positive CR-CSCs/CICs [35]. Taken together, the results indicate that activation of the Akt pathway might have an essential role in the maintenance of CR-CSCs/CICs.

Cancer immunotherapy is one possible clinical application of STn antigen. A synthetic STn-KLH vaccine was designed and used in clinical trials from the mid-1990's to mid-2000. Although the therapeutic vaccine was safe and showed a strong effect against not only breast cancer but also colon and ovarian cancers in a phase II clinical trial [39], it failed to improve overall survival of patients with metastatic breast cancer in a phase III large randomized trial [40, 41]. However, potential miss enrollment in the clinical study design was pointed out in a review article [21]. The authors of that review article also stated that STn antigen was associated with loss of cell differentiation (cancer stem cell phenotype in this study) and that the effectiveness of the therapeutic vaccine in hormone receptor-positive patients was limited because the hormone receptor is known to be correlated with a differentiated state of cells [21]. The results of those clinical trials were negative for anti-STn therapeutic immunotherapy; however, there is still a possibility to improve the design of the clinical study. Itzkowitz et al. reported that STn antigen was also detected in premalignant lesions of inflammatory bowel diseases such as ulcerative colitis and was suspected to be associated with carcinogenesis [42]. Velazquez-Martin also reported that STn antigen was detected in normal mucosa close to adenocarcinoma lesions in colorectal cancer cases [43], suggesting that accumulation of STn antigen may be the initial step of carcinogenesis. Thus, STn antigen-targeting immunotherapy might be effective for preventing carcinogenesis in patients at high risk for cancer development (prophylactic vaccine).

In summary, we identified a CR-CSC/CIC-specific antigen, ST6GALNAC1, by cDNA microarray analysis from primary CR-CSCs/CICs. STn antigen enhanced the CSC/CIC phenotype via activation of the Akt pathway by a synergistic effect with galectin-3. Since ST6GALNAC1-positive cancers are associated with poor prognosis, targeting ST6GALNAC1 and STn antigen might be a novel treatment for preventing metastasis and recurrence of adenocarcinomas including colorectal cancer.

## MATERIALS AND METHODS

### Ethics statement

Mice were maintained and experimented on in accordance with the guidelines of and after approval by the Committee of Sapporo Medical University School of Medicine, Animal Experimentation Center under permit number 12-069. Any animal found unhealthy or sick was promptly euthanized. All studies were approved by the Institutional Review Board (IRB) of Sapporo Medical University Hospital. Written informed consent was obtained from all patients according to the guidelines of the Declaration of Helsinki.

### Isolation of CR-CSCs/CICs from human primary colorectal cancer tissues

Primary CRC samples were obtained with the approval of the ethics committee from surgically resected specimens of patients who underwent operations in Sapporo Medical University Hospital. Sample tissues were collected from both cancer mucosa and normal mucosa and were then washed carefully in phosphate buffered saline (PBS) several times. A few pieces of cancer and normal mucosal tissues were stored at −80°C for future analyses. Cancer tissues were minced with scalpels and incubated in Dulbecco›s modified Eagle›s medium (DMEM) (Thermo Fisher Scientific, Yokohama, Japan) containing 0.2 mg/ml Liberase^TM^ (Roche, Basel, Switzerland) at 37°C for 30–60 mins. A single cell suspension was collected and tissue debris was removed by using a 70 μm cell strainer. The cells were washed once with PBS and red blood cells were hemolyzed by BD Pharm Lyse™ Lysing Buffer (BD biosciences) for 5 mins. Then the cells were washed again and cultured in two conditions: one condition was an adherent culture using DMEM supplemented with 10% fetal bovine serum (FBS)(GE, and Biosera), and the other condition was a sphere culture using DMEM/F12 (Thermo Fisher Scientific) supplemented with 20 ng/ml recombinant human FGF basic (R&D Systems), 20 ng/ml recombinant human EGF (R&D Systems) ×100 sodium pyruvate (Thermo Fisher Scientific), N2 supplement (Thermo Fisher Scientific) and holo transferrin (Wako) in an ultra-low attachment plate. Spheroids were treated with 0.05% trypsin-0.5 M EDTA-PBS every 3 or 4 days.

### RT-PCR and real-time quantitative RT-PCR (qRT-PCR)

Total RNA samples were extracted using an RNeasy Mini Kit (QIAGEN), and cDNA samples were synthesized with 2 μg of total RNA using Superscript III reverse transferase (Thermo Fisher Scientific) according to the manufacturer's instructions. The reaction was performed using Taq DNA polymerase (QIAGEN) and thermal cycling conditions were as follows: initial denaturation for 2 min at 94°C followed by 35 cycles of denaturation for 15 sec at 94°C, annealing for 30 sec at 58°C, elongation for 30 sec at 72°C and final elongation for 5 min at 72°C. Amplification products were applied on a 1.5% agarose gel, separated by electrophoresis, and visualized by a Dolphin-View image system (Wealtec, Sparks City, Nevada, USA). GAPDH was used as an internal positive control. The primers used in experiments are summarized in Supplementary Table 1.

Quantitative RT-PCR (qRT-PCR) was performed using an ABI PRISM 7000 Sequence Detection System (Thermo Fisher Scientific) according to the manufacturer's instructions. *ALDH1A1*, *NANOG*, *POU5F1*, *SOX2*, *LGR5* and *ST6GALNAC1* probes were designed by the manufacturer (TaqMan Gene expression assays; Applied Biosystems), and thermal cycling was performed under the following conditions: 45 cycles of 95°C for 15 sec followed by 60°C for 1 min. Detection of *LGALS3* was performed by SYBR-Green (Thermo Fisher Scientific). Each experiment was done in triplicate, and GAPDH was used as internal normalization.

### Sphere-forming assay

A sphere-forming assay was performed as described previously [44]. Cells were treated with 0.05% trypsin-0.5M EDTA-PBS and washed with PBS, and then 1.0 × 10^2^−1.0 × 10^4^ cells were seeded in 3 ml of the sphere culture medium in an ultra low attachment multi-well plate (6-well plate, Corning) and numbers of tumor spheroids were counted at day 4 or day 7 under an optical microscope using Lumina vision software (Mitani Corp.). For inhibition of Akt, the Akt inhibitor AZD5363 (15406, Cayman Chemical, MI, USA) was used at 3 μM. We excluded tumor spheroids smaller than 100 μm as a counting standard. Each experiment was done in triplicate.

Limiting dilution analysis was performed for estimation of CSC/CIC ratios. Serially diluted ST6GALNAC1 siRNA-transfected CRC21 cells and HCT116 cells were seeded into a 96-well Ultra-Low Attachment Plate (Corning^®^) in a sphere-forming medium for 14 days. Then sphere-forming wells were counted and estimated ratios of CSC/CIC were calculated at the ELDA web site (http://bioinf.wehi.edu.au/software/elda/) [45].

### Xenograft transplantation

All animal experiments were conducted according to the ethical guidelines for animal experiments of the institutions by the Ministry of Health, Labor and Welfare, Japan. Five to six-week-old nonobese diabetic/severe combined immunodeficiency (NOD/SCID) mice (NOD.CB17-Prdkcscid/J, Charles River Laboratory, Yokohama, Japan) and NOD SCID gamma (NSG) mice were used. Single cell suspensions were resuspended in 100 μl of PBS at a density of 2.0 × 10^2^−2.0 × 10^4^ cells/100 μl and mixed with 100 μl of matrigel matrix (Corning) just before injection. Half of the mixture (1.0 × 10^2^−1.0 × 10^4^ cells) was injected subcutaneously in the middle backspace of each of the recipient mice under anesthesia. Xenografted tumors were measured every week until 7 weeks after injection, and external tumor volume was calculated by the following formula: Volume (mm^3^) = Length × Width^2^ / 2.

### Resistance to 5-fluorouracil (FU)

Four thousand cells were seeded and incubated in 100 μl of culture medium containing 5-FU (Kyowa Kirin, Tokyo, Japan) at concentrations of 0, 0.2, 0.4, 0.6, 0.8, and 1.0 μg/ml in a multi-well plate (96-well plate, Corning) for 4 days, and then 10 μl of WST-1 solution (Dojindo, Kumamoto, Japan) or WST-8 (Cell Counting Kit-8, Dojindo) was added to each sample and incubated for 4 hours at 37°C in a CO_2_ incubator. Absorbance of each sample was measured using an iMark microplate absorbance reader (Bio-Rad) at dual wavelengths (measurement: 445 nm, reference: 690 nm). Each experiment was done in triplicate and proliferation rate was calculated by following the formula: Proliferation (%) = (A_sample_-A_blank_)/(A_control_-A_blank_) × 100.

### Aldefluor assay and flowcytometry

An aldefluor assay was performed using an Aldefluor Kit (STEM CELL Technologies, Tokyo, Japan) as described in the manufacturer's protocol. Collected cells were resuspended at a density of 1.0 × 10^6^ cells/ml in Aldefluor assay buffer. Propidium iodide (PI) was added to stain dead cells just before analysis to exclude dead cells. Each sample was analyzed by FACS aria II (BD Biosciences). An FITC-positive gate was fixed using a negative control sample incubated with DEAB. CSCs/CICs (FITC^high^/SSC^low^) and non-CSCs/CICs (FITC^low^/SSC^low^) were collected by cell sorting.

For detection of cell surface CD44 and CD133, an anti-CD44 antibody (ThermoFisher SCIENTIFIC, #11-0441-82) and an anti-CD133 (Milteny Biotech, 130-090-853) were used. The stained cells were analyzed by FACS aria II.

### Total RNA isolation and microarray preparation

We use two databases to identity CSC/CIC-specific genes: one was a database for transcriptional profiling of sphere-cultured cells compared with adherent-cultured cells and the other was a database for ALDH^high^ cells compared with ALDH^low^ cells. Total RNA was isolated from collected cells using an RNeasy Mini Kit (QIAGEN, Valencia, CA) following the manufacturer's instructions. We used the commercially available Low Input Quick Amp Labeling Kit (Agilent Technologies), and purified total RNA (3 μg) was reverse-transcribed to generate double-stranded cDNA using an oligo dT T7 promoter primer and reverse transcriptase. cRNA was synthesized using T7 RNA polymerase, which simultaneously incorporated Cy3- or Cy5-labeled cytidine triphosphate. The quality of cRNA was again checked using Nano Drop. Cy3-labeled cRNA and Cy5-labeled cRNA were combined and then fragmented using a gene expression hybridization kit (Agilent Technologies). Then the labeled cRNAs were hybridized to a 60-mer probe oligonucleotide microarray (GPL13497 Whole Human Genome Microarray 4 × 44 K V2) and incubated for 20 hours at 50°C. The fluorescent intensities were determined by an Agilent Technologies Scanner G2505C. Microarray raw data and processed data have been deposited in the NCBI GEO database (GSE77149 and GSE77150).

### Western blotting and immunoprecipitation

Protein samples were applied to SDS-PAGE, and separated proteins were transferred onto a PVDF membrane (Immobion-P transfer membrane, Melck) at a constant current for 60 min. After blocking with 5% skim milk in tris-buffered saline containing 0.03% Tween20 (TBS-T) for 40 min, the membrane was incubated with a primary antibody at a dilution of 1:1000 (1:2000 for β-actin) in TBS-T containing 5% skim milk for 40 min at room temperature and overnight at 4°C. The membrane was washed with TBS-T 3 times and incubated with a secondary antibody (Cat # 074-1516 (anti-rabbit) and 074-1806 (anti-mouse), KPL) diluted at 1:1000 in TBS-T containing 5% skim milk for 60 min (30 min for β-actin and B72.3). After washing the membrane again, protein detection was performed by using an ODYSSEY Fc imaging system (LI-COR) with an Amersham ECL Western blotting detection reagent (GE Healthcare). Primary antibodies used were anti-ST6GALNAC1 (HPA014975, Atlas Antibodies), anti-B72.3 as an anti-STn antigen (sc-20042, Santa Cruz), anti-pan-Akt (#4691S, Cell Signaling), anti-Phospho-Akt (#4060S, Cell Signaling), anti-S6 (#2217, Cell Signaling), anti-S6 anti-phospho-S6 (Ser325/236) (#2211, Cell Signaling) anti-β-catenin (GTX101435, Gene Tex) and anti-β-actin (A5316, Sigma-Aldrich).

Western blots for detection of SOX2 and β-Actin in CRC21 cells transfected with ST6GALNAC1 siRNA were analyzsed by the WES system (ProteinSimple ,San Jose, CA). All procedures were performed according to the manufacturer›s recommendations using the supplied agents. Anti-SOX2 antibody and anti-β-Actin were used at 50-times dilution. The resulting data were analyzed using Compas software (ProteinSimple).

Immunoprecipitation of STn antigen was performed using an immunoprecipitation kit (Abcam, ab206996) as described in the manufacturer's protocol. Briefly, 10^6^ of wild-type CRC21 cells (CRC21 w/t) and ST6GALNAC1-overexpressed cells samples 1 and 2 (overexpressed 1 and overexpressed 2) were lysed with lysis buffer. Then the cell lysates were used for immunoprecipitation using 1 μg of anti-STn antigen (clone: B72.3, sc-20042, Santa Cruz). The immunoprecipitates were analyzed by silver staining using a Silver Stain MS Kit (WAKO) as described in the manufacturer's protocol. The ST6GALNAC1-specific band around 130 kDa was cut and analyzed by TOF/TOF-MS 4800 Plus MALDI TOF/TOF Analyzer (AB SCIEX, Ontario, Canada). The immunoprecipitates were analyzed by Western blots using an anti-STn antibody (sc-20042, Santa Cruz) and an anti-CD44 antibody (GeneTex, GTX131669).

### Immunohistochemical (IHC) staining

Immunohistochemical staining of human colorectal cancer tissues was performed using an anti-ST6GALNAC1 antibody (HPA014975, Atlas Antibodies) as described previously [46]. The anti-ST6GALNAC1 antibody was used at 1000-times dilution. The positive rates of ST6GALNAC1 ranged from 0% to 80%. Cases with a positive rate of 0% were grouped as ST6GALNAC1-negative cases and ST6GALNAC1 5–80% cases were grouped as ST6GALNAC1-positive cases.

### Small interfering RNA (siRNA) transfection

Predesigned siRNAs of ST6GALNAC1 and LGALS3 (galectin-3) were purchased (Stealth siRNA; HSS125080, HSS125081 and HSS183287 for ST6GALNAC1 and HSS180668, HSS180669 and HSS180670 for LGALS3, Invitrogen), and siRNA transfection was performed using Lipofectamine RNAimax reagent (Invitrogen) as described in the manufacturer's protocol. Cells were seeded at a density of 5.0 × 10^4^ cells/ml in an antibiotic-free culture medium (DMEM, Invitrogen) supplemented with 10% FBS before transfection. Negative control Med GC duplex (Invitrogen) was used as a negative control. Transfected cells were harvested 48–72 hours after transfection and used for serial experiments.

### DNA transfection and isolation of ST6GALNAC1-overexpressed cells

ST6GALNAC1 encoding cDNA [20] was transfected by using a Neon Transfection System (Invitrogen) or Lipofectamine 3000 transfection kit (Invitrogen) according to the manufacturer's protocol. Transfected cells were maintained in a culture medium and selected by 0.6 mg/ml Geneticin (Invitrogen). Protein expression of ST6GALNAC1 and STn antigen in stable transformants was confirmed by Western blots. STn antigen-overexpressed clones were established by single cell sorting by FACS Aria II using FITC-labeled anti-Sialyl Tn antibody (ab76756, Abcam).

### Statistical analysis

Data are presented as means ± SE or SD. Statistical difference between two groups was determined by Student's *t*-test (two-tailed test). Each experiment was done in triplicate or three times. Kaplan-Meyer analysis was used to estimate survival curves, and the difference between two groups was determined by the log-rank test. *P* values < 0.05 were considered to be significant (^*^P < 0.05, ^**^P < 0.005). Statistical analysis was done with BellCurve for Excel for Windows (Social Survey Research Information Co., Ltd.)

## SUPPLEMENTARY MATERIALS FIGURES AND TABLE


