# Supplementary material for: ST6GALNAC1 plays important roles in enhancing cancer stem phenotypes of colorectal cancer via the Akt pathway
Source: Oncotarget. 2017 Nov 8;8(68):112550–64. doi: 10.18632/oncotarget.22545 (PMC5762531; doi:10.18632/oncotarget.22545)
Supplement: Supplementary file 1 [file oncotarget-08-112550-s001.pdf]

ST6GALNAC1 plays important roles in enhancing cancer stem phenotypes of colorectal cancer via the Akt pathway

SUPPLEMENTARY MATERIALS

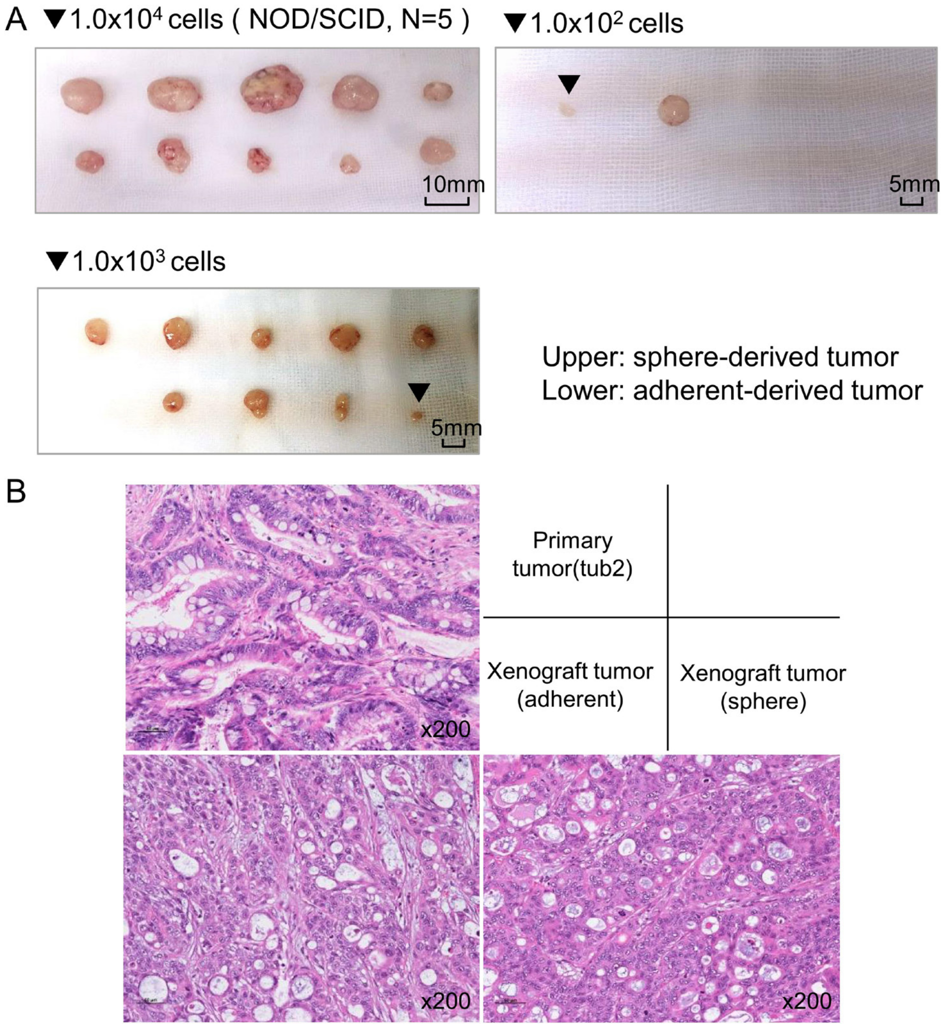

Supplementary Figure 1: Characterization of human primary CR-CSCs/CICs (CRC21 cells). (**A**) Xenografted tumors. Tumor imaged derived from xenografted mouse using 1×10^2, 1×10^3 and 1×10^4 cells. Sphere-cultured CRC21 cells (upper) and adherent-cultured CRC21 cells (lower) were used. (**B**) Histologies of primary tumor and xenografted tumors (adherent-cultured and sphere-cultured cells). Tumors were stained by hematoxylin and eosin (HE). Magnification, ×200.

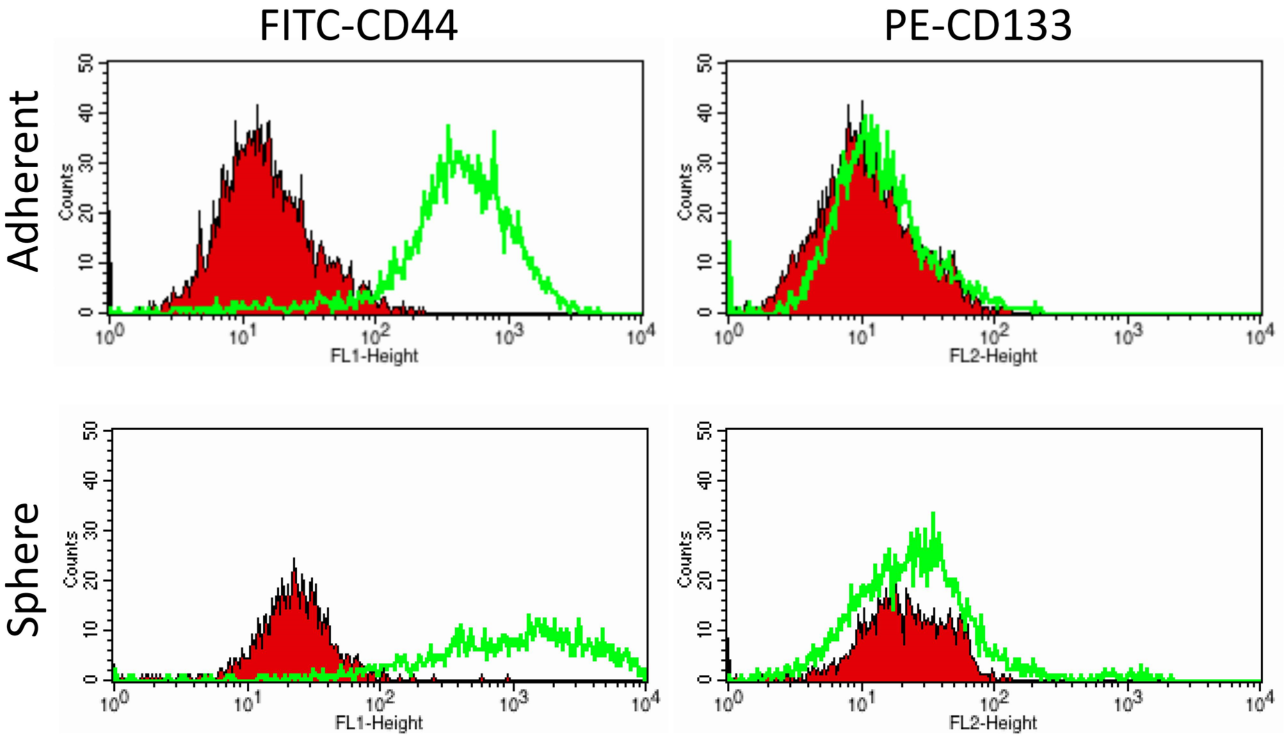

Supplementary Figure 2: The expressions of CD44 and CD133 in sphere-cultured cells. CRC21 cells were cultured in sphere-culture condition and adherent-condition for 7 days, then analyzed the expressions of CD44 and CD133 by flow cytometry. Red indicate negative controls and green indicate CD44 or CD133 stained sample.

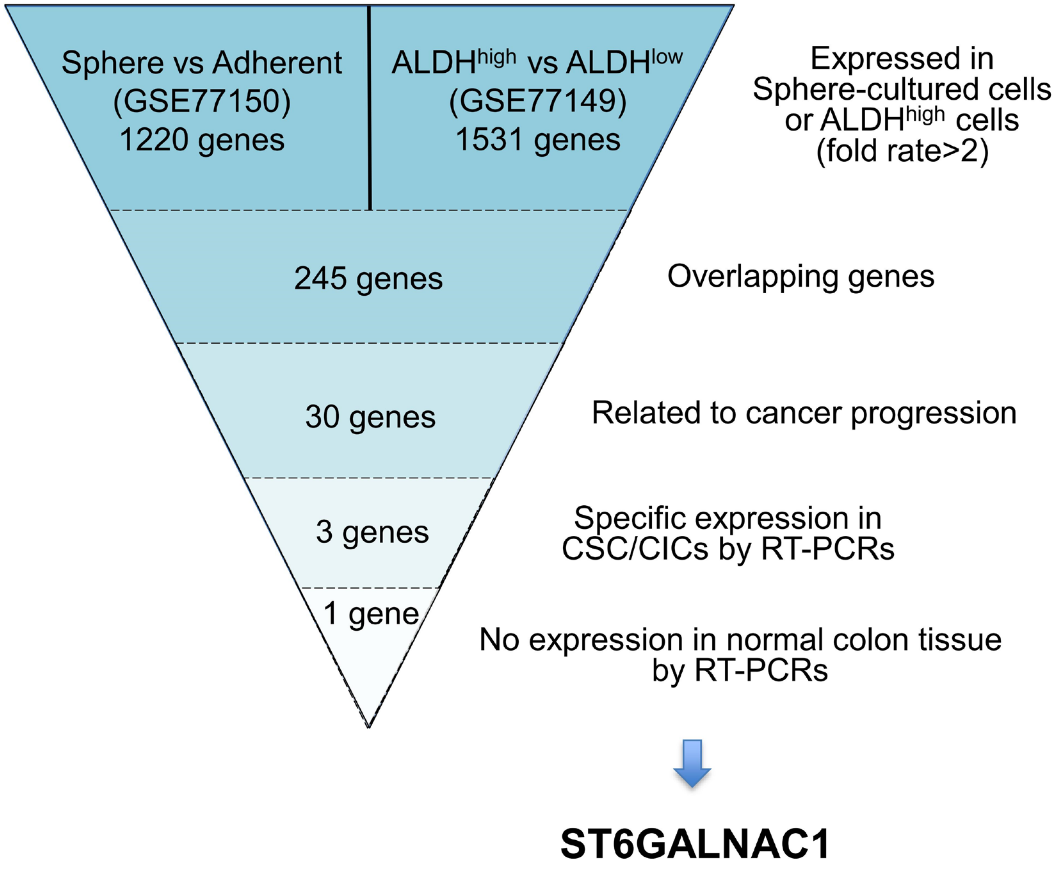

Supplementary Figure 3: Schematic summary of narrowing down CSC/CIC-specific genes by two microarray analysis.

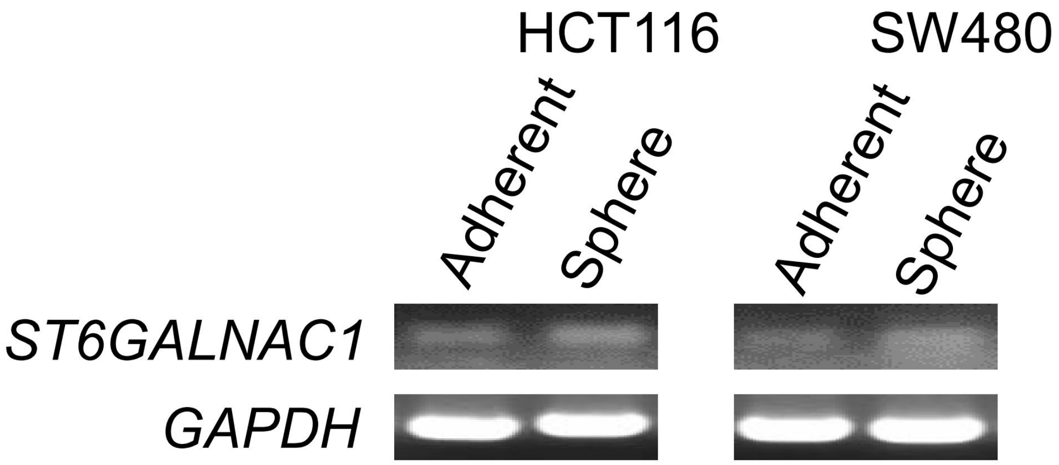

Supplementary Figure 4: Expression of ST6GALNAC1 in sphere-cultured HCT116 cells and SW480 cells. RT-PCRs were performed using cDNAs derived from adherent-cultured and sphere-cultured HCT116 cells and SW480 cells. GAPDH was used an internal control.

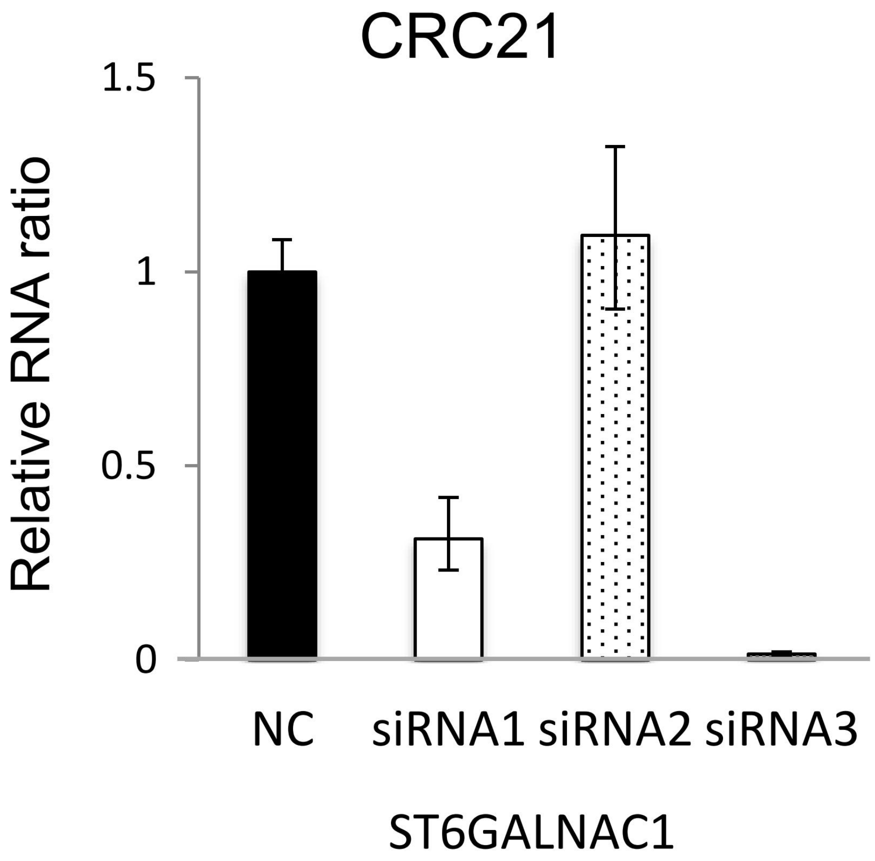

Supplementary Figure 5: ST6GALNAC1 knockdown by siRNAs. Three different siRNA and negative control siRNA were transfected into CRC21 cells and expression level of ST6GLNAC1 in each sample was analyzed using qRT-PCR. Data are shown as means ± SD.

Stem cell frequency of ST6GALNAC1 knocked down cells							
HCT116 cells	sphere-positive wells				CSC frequency	95% CI	†P value
	1 cell /well	10 cells /well	100 cells /well	1000 cells /well			
N/C	0/24	1/24	7/24	24/24	1 in 240	146-393	-
siRNA1	0/24	0/24	3/24	20/24	1 in 603	388-939	0.00676*
siRNA3	0/24	0/24	0/24	19/24	1 in 803	507-1271	0.000484*

†Defference with cells transfected with negative control siRNA were caliculated by Chi-square test. *P<0.05

Supplementary Figure 6: Stem cell ratios in ST6GALNAC1-knockdown cells. HCT116 cells transfected with siRNAs (control, ST6GALNAC1 siRNA1, ST6GALNAC1 siRNA3) were seeded into a 96-well plate at 1, 10, 100 and 1000 cells/well under a sphere-forming condition and cultured for 7 days. The sphere-positive wells were counted and are shown. Stem cell ratios in negative control cells and ST6GALNAC1-knockdown cells were calculated by the ELDA website.

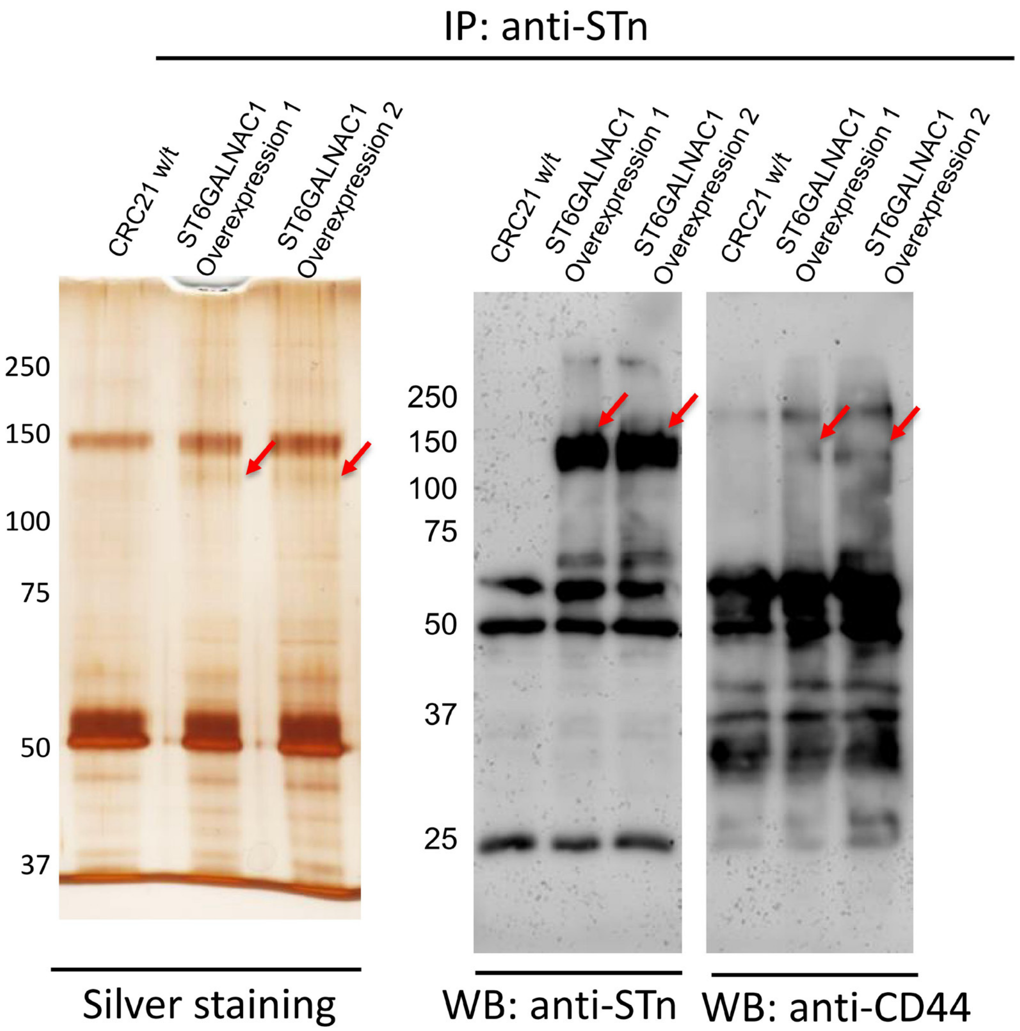

Supplementary Figure 7: Analysis of STn antigen expressed in ST6GALNAC1 overexpressed cells. ST6GALNAC1 gene was overexpressed in CRC21 cells (overexpression 1 and 2). The cell lysates of CRC21 w/t cells and ST6GALNAC1 overexpressed cells were immunoprecipitated using anti-STn antibody. The precipitates were analyzed by silver staining, and Western blotting using anti-STn antigen and anti-CD44 antibody. Numerical indicate molecular weights (kDa). Red arrows indicate ST6GALNAC1 specific bands.

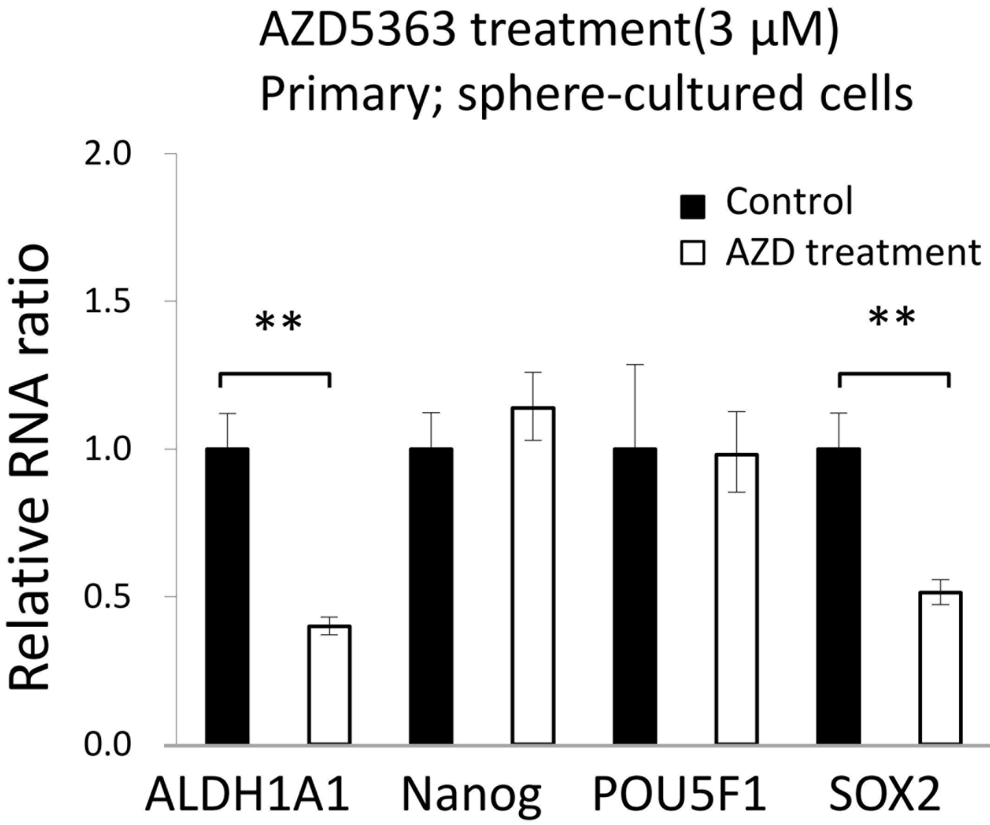

Supplementary Figure 8: Inhibition of Akt inhibitor decreased the expressions of ALDH1A1 and SOX2. Sphere-cultured cells were treated by an Akt inhibitor AZD5363 (3 µM), then the expressions of stem cell related genes were analyzed by qRT-PCR. Data are shown as means ± SD.

Supplementary Table 1: Primer sequences for RT-PCR

Gene Name		Primer sequence (5′→3′)	Product size (bp)
ALDH1A1	forward	TGTTAGCTGATGCCGACTTG	153
	reverse	TTCTTAGCCCGCTCAACACT	
Nanog	forward	GCTGAGATGCCTCACACGGAG	163
	reverse	TCTGTTTCTTGACCGGGACCTTGTC	
POU5F1	forward	TGGAGAAGGAGAAGCTGGAGCAAAA	186
	reverse	GGCAGATGGTCGTTTGGCTGAATA	
SOX2	forward	CATGATGGAGACGGAGCTGA	420
	reverse	ACCCCGCTCGCCATGCTATT	
ST6GALNAC1	forward	TCTCCCTGACCCAGTCACTC	198
	reverse	CTTCCCGAAAAGCTTCCTG	
LGALS3	forward	TGGAGCACCTGGAGCTTATC	221
	reverse	CCGTGCCCAGAATTGTTATC	
GAPDH	forward	ACCACAGTCCATGCCATCAC	452
	reverse	TCCACCACCCTGTTGCTGTA	
